# The Process of Magnetizing FeNbYHfB Bulk Amorphous Alloys in Strong Magnetic Fields

**DOI:** 10.3390/ma13061367

**Published:** 2020-03-18

**Authors:** Bartłomiej Jeż, Jerzy Wysłocki, Simon Walters, Przemysław Postawa, Marcin Nabiałek

**Affiliations:** 1 Department of Physics, Faculty of Production Engineering and Materials Technology, Częstochowa University of Technology, Al. Armii Krajowej 19, 42-200 Częstochowa, Polabd; bartek199.91@o2.pl (B.J.); wyslocki.jerzy@wip.pcz.pl (J.W.); 2 Division of Engineering School of Computing, Engineering and Mathematics, University of Brighton, BN2 4GJ Brighton, UK; S.D.Walters@brighton.ac.uk; 3 Department of Technology and Automation, Faculty of Mechanical Engineering and Computer Science, Częstochowa University of Technology, Al. Armii Krajowej 21, 42-200 Częstochowa, Poland; postawa@ipp.pcz.pl

**Keywords:** bulk metallic glasses, H. Kronmüller theory, Holstein–Primakoff paraprocess

## Abstract

The structure of amorphous alloys still has not been described satisfactorily due to the lack of direct methods for observing structural defects. The magnetizing process of amorphous alloys is closely related to its disordered structure. The sensitivity of the magnetization vector to any heterogeneity allows indirect assessment of the structure of amorphous ferromagnetic alloys. In strong magnetic fields, the magnetization process involves the rotation of a magnetization vector around point and line defects. Based on analysis of primary magnetization curves, it is possible to identify the type of these defects. This paper presents the results of research into the magnetization process of amorphous alloys that are based on iron, in the areas called the approach to ferromagnetic saturation and the Holstein–Primakoff para-process. The structure of a range of specially produced materials was examined using X-ray diffraction. Primary magnetization curves were measured over the range of 0 to 2 T. The process of magnetizing all of the tested alloys was associated with the presence of linear defects, satisfying the relationship D_di p_ < 1_H_. It was found that the addition of yttrium, at the expense of hafnium, impedes the magnetization process. The alloy with an atomic content of Y = 10% was characterized by the highest saturation magnetization value and the lowest value of the D_spf_ parameter, which may indicate the occurrence of antiferromagnetic ordering in certain regions of this alloy sample.

## 1. Introduction

Ferromagnetics can be classified according to their ease of magnetization, where the classification criterion is the value of the coercive field. In the case of a coercive field value of less than 1000 A/m, it is assumed that the associated materials are ‘soft magnetic’; in the range from 1000–10,000 A/m, ‘semi-hard’; and if greater than 10,000 A/m, ‘hard’ [1]. Magnetically hard properties are exhibited by permanent magnets. Materials with semi-hard magnetic properties are used, for example, in the construction of magnetic memory systems. Due to their ease of magnetization (and re-magnetization), alloys with soft magnetic properties are widely used in electronics and electrical engineering applications; for example, they can be used in the construction of low-loss transformer cores [2,3].

The amorphous iron-based alloys are commonly known materials with soft magnetic properties. Among these materials, alloys produced by rapid quenching are most popular. Materials that are produced in the form of thin ribbons, using the melt-spinning method [4], present particularly good properties. Materials of this type are characterized by a high value of saturation magnetization (above 1.5 T) and a low value of coercive field (approximately 1 A/m) [5,6,7,8,9]. These material samples are extremely easy to magnetize, partly due to their dimensions. Amorphous ribbons have thicknesses of up to several tens of µm. Unfortunately, these dimensions significantly limit the applications of these materials. The so-called bulk amorphous alloys comprise a relatively new group of promising materials. These materials are produced by a rapid-quenching process in copper molds. The most well-known production methods are the injection- and suction-casting methods [10,11]. In this way, at a cooling rate of 10^−1^–10^3^ K/s, iron-based alloys with dimensions exceeding 10 mm can be produced [12,13,14]. However, due to their high proportions of non-magnetic component elements, such as: B, C, Zr, Y, Mo, Nb, Hf, or Cr, these alloys do not exhibit the best magnetic properties. A compromise that combines relatively good magnetic properties (saturation magnetization greater than1T, coercive field less than 50 A/m) with favorable alloy geometry (diameter up to 3 mm) exists in the form of alloys with a content of approximately 65%–75% magnetic elements [15,16,17,18].

**Figure 1:**
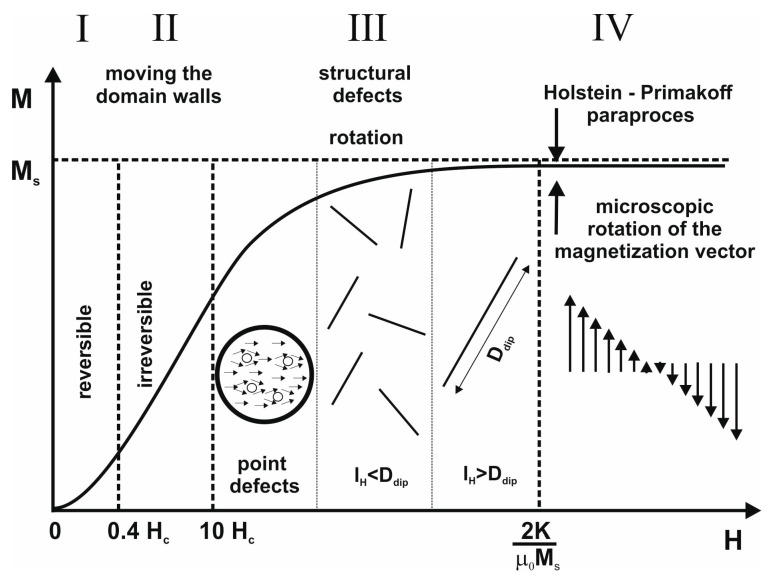
The curve of primary magnetization divided into characteristic areas [19].

The process of magnetizing amorphous alloys does not differ significantly from their crystalline counterparts. Figure 1 shows a diagram of the primary magnetization curve. 

In the first area, magnetization is associated with reversible shifts of domain walls. Above 0.4 H_C_, domain wall shifts are irreversible. An increase in the intensity of the external magnetic field causes the disappearance of magnetic domains with directions that do not correlate with the applied direction of the magnetic field. In the third area, closing domains are magnetized. In the case of materials with an amorphous structure, magnetization in this area is associated with the presence of structural defects—occurring in the form of free volumes and pseudodislocation dipoles [20,21,22]. As the applied magnetic field increases, the magnetization vector rotates around point, and then linear, defects. In the fourth area, further magnetization of the alloy is associated with the damping of thermally excited spin-waves; i.e., the so-called Holstein–Primakoff paraprocess [23].

As part of this work, rapidly quenched alloys with the chemical compositions: Fe_65_Nb_5_Y_5+x_Hf_5−x_B_20_ (where: x = 0, 1, 2, 3, 4, 5) were produced, using the injection-casting method. An analysis of the magnetization process in high magnetic fields was carried out; i.e., within the third and fourth regions of the primary magnetization curve. The aim of the study was to determine the effect of Y and Hf content on the magnetization process of bulk Fe-based amorphous alloys.

## 2. Materials and Methods 

Polycrystalline ingots, each weighing 10 grams, were made using an arc furnace. The purities of the ingredients were as follows: Fe = 99.99% at, Hf = 99.98% at, Nb = 99.98% at, Y = 99.98% at, and B = 99.98% at. Boron was added in the form of a pre-prepared FeB alloy with the chemical composition Fe_45.4_B_54.6_. The production process was carried out under a protective atmosphere of argon. The charge was melted by plasma arc using a current of 180–380 A, flowing through a non-melting tungsten electrode. The ingots were melted on a water-cooled copper plate. Each remelting of the charge was preceded by the remelting of a titanium getter. The ingots were remelted four times on each side. The resulting alloys were mechanically cleaned, divided into smaller pieces, and cleaned again using an ultrasonic cleaner. Rapid-quenched alloys were made using the injection-casting method. The polycrystalline charge was placed in a quartz crucible inside a copper coil. The charge was melted using eddy currents at a constant current of 10 A. The production process was carried out in a protective atmosphere of argon. The liquid alloy was forced into a water-cooled copper mold; 0.5mm-thick plates were made.

The structure of the produced alloys was examined using X-ray diffraction. Samples, in the form of powder, were irradiated for 6 seconds per measuring step (0.02°). A Bruker D8 Advance X-ray diffractometer (Bruker, Billerica, MA, USA), equipped with a CuKα lamp and a semiconductor meter, was used; the measurement was carried out in the range of 30–100º of the two-theta angle.

The microstructure of each of the alloys was investigated using a POLON Mössbauer spectrometer, the latter being equipped with a ^57^Co radioactive source of activity 100 mCi and half-life of 270 days. Calibration of the spectrometer was performed by recording the spectrum for an α-Fe foil with a thickness of 20 µm. For all the prepared and measured samples of the studied alloys, the surface density of the 57Fe isotope was less than 0.3 mg/cm^2^. This allowed the use of the “thin absorbent” approximation. Analysis of the transmission Mössbauer spectra was performed using the NORMOS software (version 3), developed by R. A. Brand [24]. This software facilitates the decomposition of the experimental spectra to the constituent spectra and the determination of the distribution of the hyperfine field induction P(B). In order to determine the distribution of the hyperfine field distribution on the ^57^Fe nuclei, according to the Hesse-Rübatsch method [25], each experimental spectrum was presented as the sum of the elementary sextets:

(1) $$T\left(v\right)=\int_{0}^{\infty }P\left(B\right)L_{6}\left(B,v\right)\mathrm{dB}$$

where: 

P(B)—distribution of the hyperfine magnetic field induction,

L_6_ (B, v)—elementary Zeeman sextet, 

v—relative speed of source against the absorbent.

From analysis of the distribution of the hyperfine magnetic field induction, the average value of the hyperfine magnetic field induction B_hf_ was determined. Due to asymmetry in the Mössbauer spectra, during the fitting process, a linear relationship between the isomer shift (IS) and the hyperfine field induction (B_hf_) was assumed [24].

(2) $$\mathrm{IS}\left(B_{\mathrm{hf}}\right)=\mathrm{IS}\left(B_{\mathrm{hf}}^{0}\right)-\alpha \left(B_{\mathrm{hf}}-B_{\mathrm{hf}}^{0}\right)$$

where: 

B^0^_hf_ = the minimum value of the hyperfine field induction,

α = coefficient.

Due to the powdered form of the investigated samples, the element A2.5 = 2 was blocked due to the lack of magnetic anisotropy related with the sample texture. Primary magnetization curves were measured using a LakeShore vibrating sample magnetometer, in the range of external magnetic field strength extending up to 2 T (Supplementary materials). The structural and magnetic properties tests were carried out at room temperature.

The primary magnetization curves were subjected to numerical analysis, according to the H. Kronmüller theory. According to the modified micromagnetism theory of Brown, (Brown’s micromagnetic equations) magnetization in high magnetic fields can be expressed approximately by the formula [20]:

(3) $$\mu_{0}M(H)=\mu_{0}M_{s}\left[1-\frac{a_{1/2}}{{\left(\mu_{0}H\right)}^{1/2}}-\frac{a_{1}}{{\left(\mu_{0}H\right)}^{1}}-\frac{a_{2}}{{\left(\mu_{0}H\right)}^{2}}\right]+b{\left(\mu_{0}H\right)}^{1/2}$$

where: *M_S_* = spontaneous magnetization, *H* = magnetic field, *a_i_* (i = ½, 1, 2) = angular coefficients of the linear fit, which correspond to the free volume and linear defects, *µ*_0_ = magnetic permeability of a vacuum, and *b* = slope of the linear fit corresponding to the thermally-induced suppression of spin-waves by a high intensity magnetic field.

The coefficients, *a_i_* (described by Equations (4)–(6)), appearing in the expression, reduce the magnetization and are associated with the presence of structural defects. The term a_1/2_/(μ_0_H)^1/2^ in the Equation (3), related to the point defects, is described as follows:

(4) $$\frac{a_{1/2}}{{\left(\mu_{0}H\right)}^{1/2}}=\mu_{0}\frac{3}{20A_{ex}}{\left(\frac{1+r}{1-r}\right)}^{2}G^{2}\lambda_{s}^{2}{\left(\Delta V\right)}^{2}N{\left(\frac{2A_{ex}}{\mu_{0}M_{s}}\right)}^{1/2}\frac{1}{{\left(\mu_{0}H\right)}^{1/2}}$$

The terms a_1_/µ_0_H and a_2_/(µ_0_H)^2^ in Equation (3) are related to the linear defects, which internal stress field is equivalent to the field generated by the linear dislocation dipoles of the D_dip_ width, the effective Burgers vector b_eff_, and the surface density N.

If l_H_D_dip_<1, where l_H_ is the inversed exchange distance described by the equation:

(5) $$l_{H}=\sqrt{\frac{H\mu_{0}M_{S}}{2A_{ex}}},$$

the dominant role in the Equation (3) plays the term a_1_/µ_0_H equal to:

(6) $$\frac{a_{1}}{\mu_{0}H}=1.1\mu_{0}\frac{G^{2}\lambda_{s}^{2}}{{\left(1-\nu \right)}^{2}}\frac{Nb_{eff}}{M_{s}A_{ex}}D_{dip}^{2}\frac{1}{\mu_{0}H}$$

For l_H_D_dip_ > 1 the term a_2_/(µ_0_H)^2^ is dominant and can be expressed by the equation:

(7) $$\frac{a_{2}}{\mu_{0}H^{2}}=0.456\mu_{0}\frac{G^{2}\lambda_{s}^{2}}{{\left(1-\nu \right)}^{2}}\frac{Nb_{eff}}{M_{s}^{2}}D_{dip}^{2}\frac{1}{{\left(\mu_{0}H\right)}^{2}}$$

where: *A_ex_* = exchange constant, *G* = transverse elastic shear modulus, Δ*V* = the change in volume due to the occurrence of a point defect characterized by a bulk density of *N, v =* Poisson’s ratio, *λ_s_* = magnetostriction constant, *D_dip_* = pseudodislocation dipole width.

The *b* factor is associated with the damping of thermally excited spin-waves. Through the equation, this factor is associated with the spin-wave stiffness parameter *D_spf_*.

(8) $$b=3.54g\mu_{0}\mu_{B}{\left(\frac{1}{4\pi D_{spf}}\right)}^{3/2}kT{\left(g\mu_{B}\right)}^{1/2}$$

where: *k* = Boltzman’s constant, *µ_B_* = Bohr magneton, *g* = gyromagnetic factor, *T* = temperature.

Based on analysis of the primary magnetization curves, it is possible to assess the type—and in some cases the number—of structural defects. The exchange constant was determined using the relationship (9), and the width of the pseudodislocation dipole from the relationship (10). The surface density of linear defects was calculated using Equation (11).

(9) $$A_{ex}=\frac{M_{s}D_{spf}}{2g\mu_{B}}$$

(10) $$D_{dip}=\sqrt{\frac{2A_{ex}}{H_{t}M_{S}}}$$

(11) $$N_{dip}=\frac{1}{l_{H}^{2}}$$

## 3. Results

**Figure 2:**
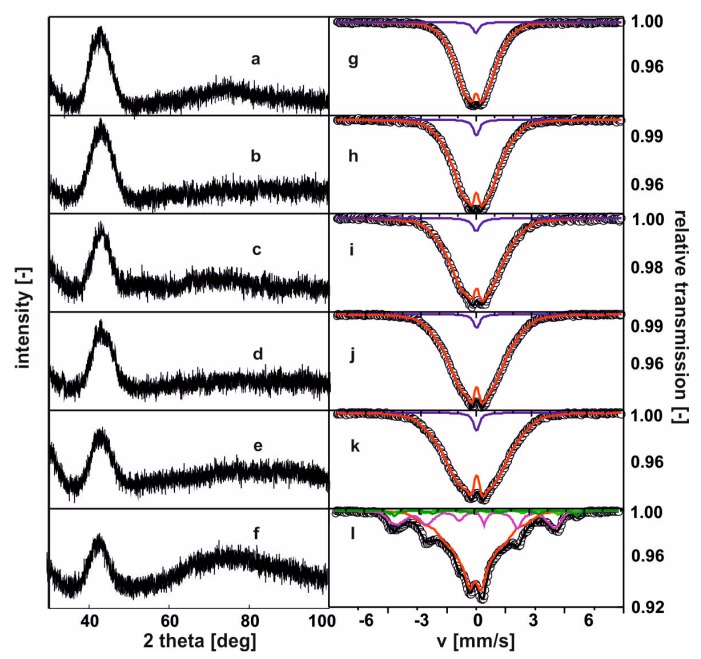
X-ray diffraction images patterns and Mössbauer spectra for the alloys: (**a**), (**g**) Fe_65_Nb_5_Y_5_Hf_5_B_20_; (**b**), (**h**) Fe_65_Nb_5_Y_6_Hf_4_B_20_; (**c**), (**i**) Fe_65_Nb_5_Y_7_Hf_3_B_20_; (**d**), (**j**) Fe_65_Nb_5_Y_8_Hf_2_B_20_; (**e**), (**k**) Fe_65_Nb_5_Y_9_Hf_1_B_20_; (**f**), (**l**) Fe_65_Nb_5_Y_10_B_20_. [26].

Figure 2 contains X-ray diffraction patterns and Mössbauer spectra measured for the tested alloys.

**Figure 3:**
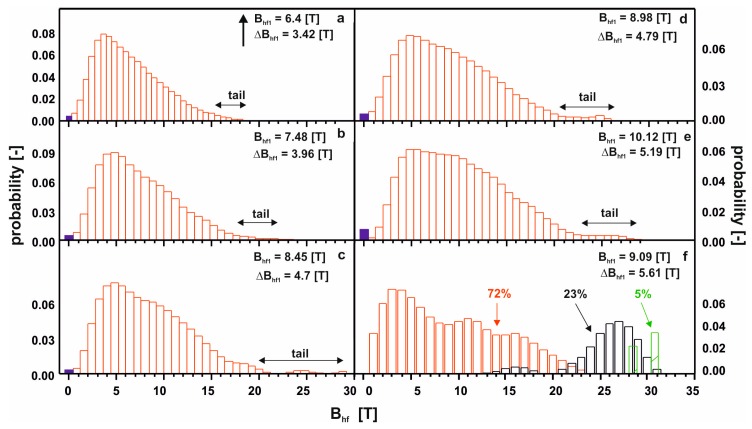
The hyperfine field distribution on the ^57^Fe nuclei for the investigated alloys: (**a**) Fe_65_Nb_5_Y_5_Hf_5_B_20_; (**b**) Fe_65_Nb_5_Y_6_Hf_4_B_20_; (**c**) Fe_65_Nb_5_Y_7_Hf_3_B_20_; (**d**) Fe_65_Nb_5_Y_8_Hf_2_B_20_; (**e**) Fe_65_Nb_5_Y_9_Hf_1_B_20_; (**f**) Fe_65_Nb_5_Y_10_B_20_.

On the recorded X-ray diffraction patterns, there were only wide maxima within the range of 40—50° of the two-theta angle. The transmission Mössbauer spectra, presented in Figure 2, were typical for ferromagnetic, amorphous alloys with a relatively low hyperfine field. In effect, low Curie temperatures are expected [26]. In the case of the first five spectra, a residual paramagnetic phase was also observed (blue line). For the last of the studied alloys, the Mössbauer spectrum consisted of three components: two resulting from amorphous matrices, and one from the minor volume of a phase with order similar to the Fe^5^Y crystalline phase. Figure 3 shows the distribution of the hyperfine field induction on the ^57^Fe nuclei, corresponding to the spectrum from Figure 2.

**Figure 4:**
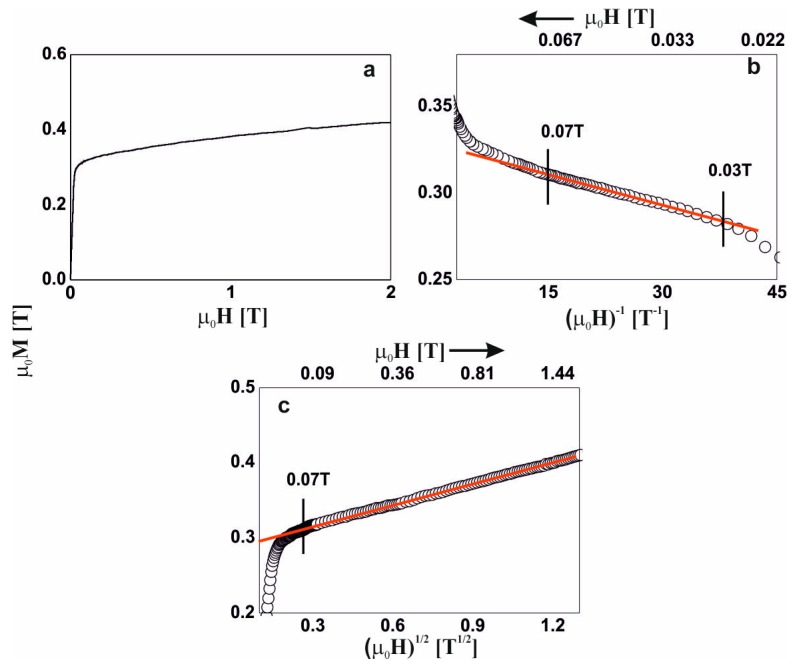
Magnetization curves for the Fe_65_Nb_5_Y_5_Hf_5_B_20_ alloy: (**a**) initial magnetization curve, magnetization as a function of: (**b**) (µ_0_H)^−1^ and (**c**) (µ_0_H)^1/2^.

**Figure 5:**
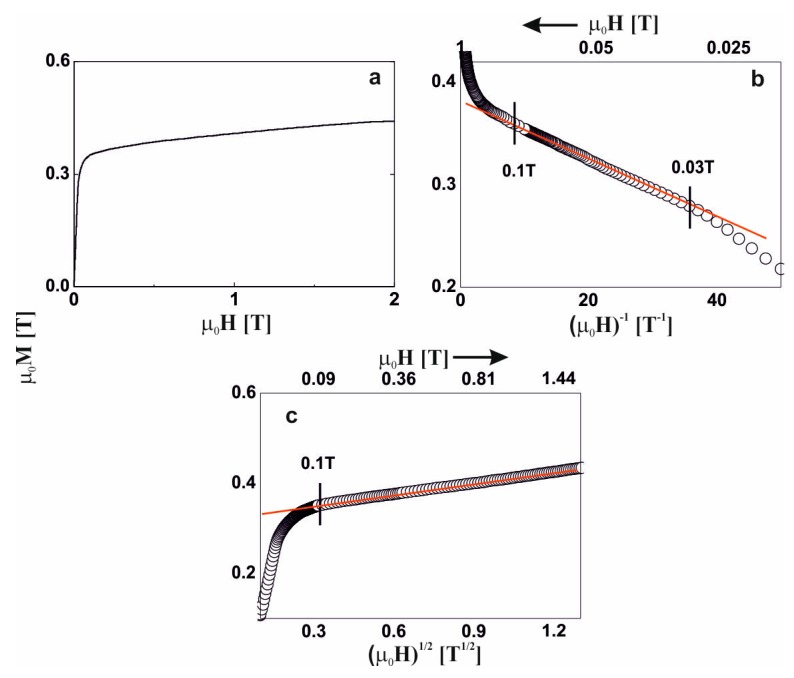
Magnetization curves for the Fe_65_Nb_5_Y_6_Hf_4_B_20_ alloy: (**a**) initial magnetization curve, magnetization as a function of: (**b**) (µ_0_H)^−1^ and (**c**) (µ_0_H)^1/2^.

**Figure 6:**
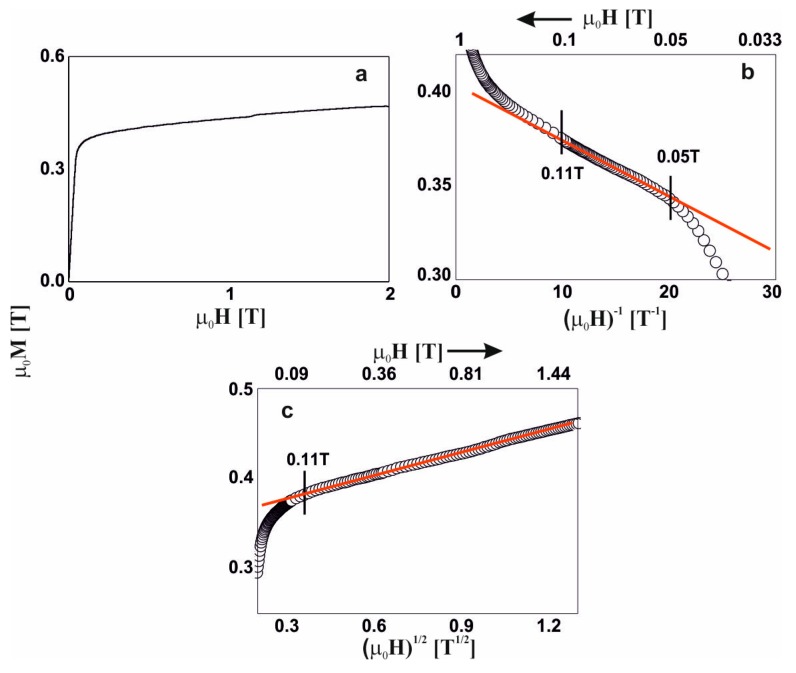
Magnetization curves for the Fe_65_Nb_5_Y_7_Hf_3_B_20_ alloy: (**a**) initial magnetization curve, magnetization as a function of: (**b**) (µ_0_H)^−1^ and (**c**) (µ_0_H)^1/2^.

**Figure 7:**
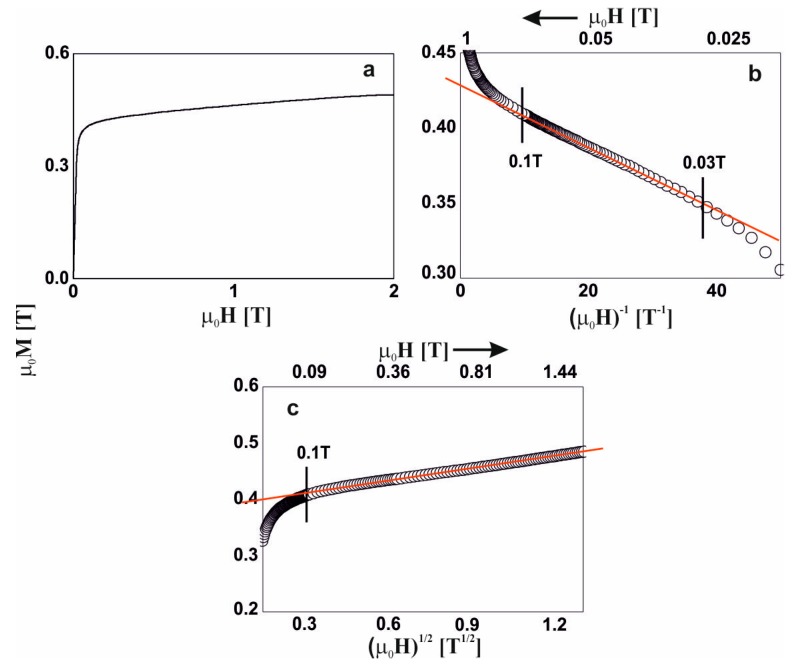
Magnetization curves for the Fe_65_Nb_5_Y_8_Hf_2_B_20_ alloy: (**a**) initial magnetization curve, magnetization as a function of: (**b**) (µ_0_H)^−1^ and (**c**) (µ_0_H)^1/2^.

**Figure 8:**
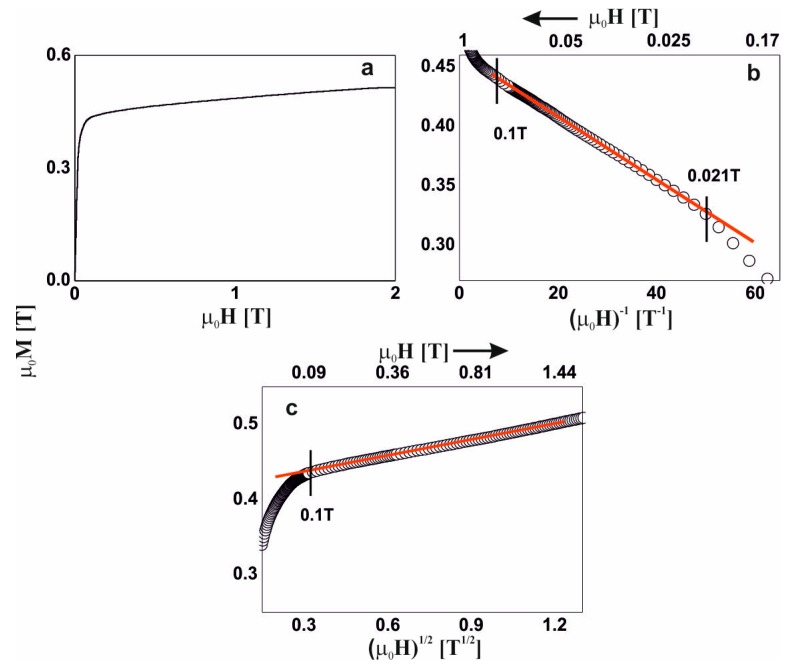
Magnetization curves for the Fe_65_Nb_5_Y_9_Hf_1_B_20_ alloy: (**a**) initial magnetization curve, magnetization as a function of: (**b**) (µ_0_H)^−1^ and (**c**) (µ_0_H)^1/2^.

**Figure 9:**
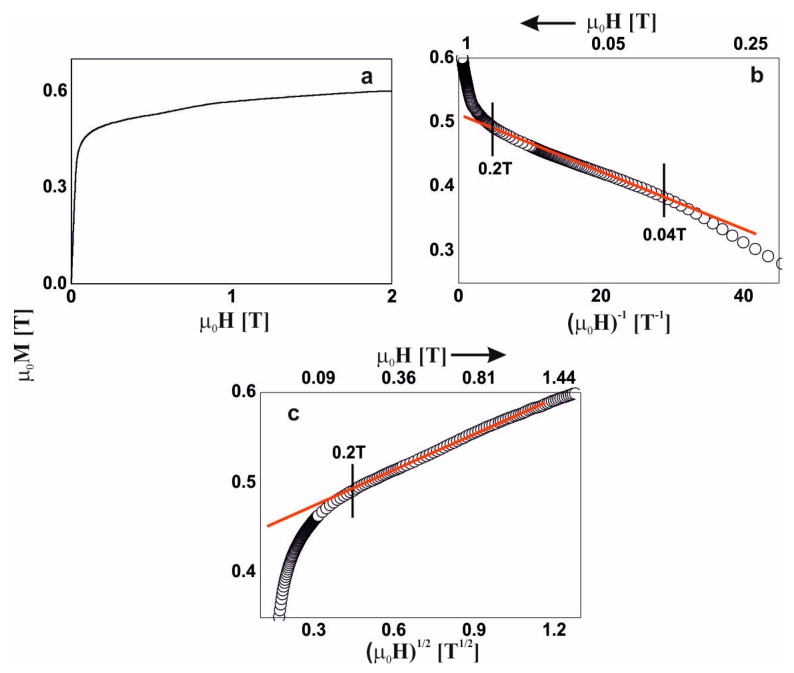
Magnetization curves for the Fe_65_Nb_5_Y_10_B_20_ alloy: (**a**) initial magnetization curve, magnetization as a function of: (**b**) (µ_0_H)^−1^ and (**c**) (µ_0_H)^1/2^.

For five of the studied alloys, in the distribution of the hyperfine field induction there was a non-zero probability of a zero value of ^57^Fe induction, which suggests the presence of a minor contribution of a paramagnetic phase (blue bar). A major content of non-magnetic components led a to significant distribution of the Fe atoms. Locally, the Fe atoms were separated to such a degree, that in some regions of the alloys, the Fe was present in paramagnetic form. The contribution of the paramagnetic phase to these samples was about 3%, and reconstitution of the distribution of hyperfine field induction was observed. In the low-field region, the probability of the presence of Fe in the vicinity of a central ^57^Fe atom was higher. Utilizing the distribution of the hyperfine fields, (2), a monotonic increase in the average value of the hyperfine field and its standard deviation were observed. With an increase in the Y content, the high-field component started to appear (in the form of a ‘tail’). The described changes for the first five alloys relate to an increase in the Y content at the expense of the Hf content. It should be mentioned that these elements had the largest atomic radii, and their exchange indirectly caused destabilization of the amorphous structure, and changes in the ferromagnetic interactions. This was confirmed by the spectrum obtained for the alloy with no Hf content. For the Fe_65_Nb_5_Y_10_B_20_ alloy, the amorphous matrix can be decomposed into three components. The amorphous structure was re-phased into two phases, with a high degree of magnetic inhomogeneity. In the case of the first magnetic phase (shown in red color), at least a tri-modal distribution can be observed with an average hyperfine field of 9.09 T; whereas for the second phase (shown in black color) the distribution is bi-modal with an average hyperfine field value of 25.7 T. Due to the substantial chemical and topological inhomogeneity of the Fe_65_Nb_5_Y_10_B_20_ alloy, an additional high-field component—described by two sextets with relatively wide lines—was isolated. The distribution of the hyperfine field obtained for these lines may suggest that they may belong to the Fe_5_Y crystalline phase [27]. However, their width is too large, and they must be treated as clustered structures—similar to the ordering of the Fe_5_Y crystalline phase. This means that the Fe_5_Y phase was in the early phase of crystallization of the high-field amorphous matrix. For all the studied alloys, the average hyperfine field value increased with increasing Y content, in addition to the dispersion of the distribution. This was related to the rebuilding of the magnetic structure. The changes in the chemical and topological order in the volume of the alloy—related to atomic diffusion—indirectly affected the degree in which the structure featured defects. According to the approach to the ferromagnetic saturation theorem of H. Kronmüller, analysis of the initial magnetization curves was performed. Figure 4, Figure 5, Figure 6, Figure 7, Figure 8 and Figure 9 present results from the analysis of the primary magnetization curves for the investigated alloys.

In the external magnetic field range of 0.03–0.07 T, the magnetization process of the Fe_65_Nb_5_Y_5_Hf_5_B_20_ alloy (Figure 4b) was associated with the rotation of the magnetization vector around linear structure defects. In magnetic fields of greater than 0.07 T, further magnetization was associated with the damping of thermally excited spin-waves (Figure 4c).

The magnetization process of Fe_65_Nb_5_Y_6_Hf_4_B_20_ alloy (Figure 5) was almost identical to the previous alloy. In this case, the Holstein–Primakoff paraprocess occurred in magnetic fields of greater than 0.1 T.

The analysis of the magnetization process of the: Fe_65_Nb_5_Y_7_Hf_3_B_20_, Fe_65_Nb_5_Y_8_Hf_2_B_20_, and Fe_65_Nb_5_Y_9_Hf_1_B_20_ alloys (Figure 6, Figure 7 and Figure 8) was similar. Linear defects, satisfying the relationship D_dip_ < 1_H_, had a dominant effect on the magnetization process in magnetic fields of less than or equal to approximately 0.1T. In the case of the Fe_65_Nb_5_Y_10_B_20_ alloy (Figure 9), based on analysis of the primary magnetization curves, the presence of linear defects—whose dimensions did not exceed the exchange distance l_H_—was also discovered.

Based on analysis of the primary magnetization curves, the following parameters were determined:

- Slope of the linear-fit curve for linear defects a_1_ [10^−3^];
- The transition area to the Holstein–Primakoff paraprocess H_a1_/H_H-P_ [T];
- Coefficient of linear fit to the Holstein–Primakoff paraprocess b [-];
- Spin-wave stiffness parameter D_spf_ [10^−2^ meVnm^2^],
- Surface density of linear defects N_dip_ [10^17^ m^−2^];
- Saturation magnetization M_S_ [T].

Table 1 contains the results of analysis of the magnetization processes pertaining to the tested alloys.

## 4. Discussion

All of the tested materials had a disordered structure, as evidenced by the recorded diffractograms; only broad, fuzzy maxima were present. These reflections arose due to the reflection of X-rays from chaotically spaced atoms within the sample volume. There were no narrow peaks that would indicate the presence of crystalline phases. All of the tested alloys were characterized by low values of saturation magnetization (0.42–0.6 T) in relation to the significant iron content (65% atomic). In the volume of the investigated alloys, it is possible that there were areas for which antiferromagnetic ordering was more privileged in energy terms. It is also possible that the tested materials had residual quantities of paramagnetic phases according with the Mössbauer results. In the case of a small number of these phases, it was not possible to identify them using X-ray diffraction. 

The value of the coercive field H_C_ [A/m] was determined on the basis of static magnetic hysteresis loops. 

For the sample Fe_65_Nb_5_Y_10_B_20_, we observed a definitely higher value of the coercive field than for the other tested alloys. The introduction of an additional component into the alloy characterized by a large atomic radius and a large negative heat of mixing in relation to most of the alloy components caused a slowdown in the movement of atoms during the solidification process, which was in accordance with the Inoue criteria. This choice of alloy composition significantly increased melt viscosity and reduced diffusion of alloying elements during solidification. On this basis, it can be assumed that the incorporation of any component into an alloy that meets the Inoue criteria will have a similar effect on glass-forming ability as Hf. As is known, amorphous materials based on Fe due to the lack of long-range interactions between atoms exhibit good soft magnetic properties. For a smaller number of components, i.e., for the FeNbYB alloy, it follows that the setting time should be longer than for the five-component FeNbHfYB alloys. This means that privileged system clusters are much easier to form in four-component systems. In numerous studies [28] of alloys with the addition of Y (FeCoYB), it has been demonstrated that one of the most frequently formed crystalline phases is magnetically soft phase αFe, magnetically semi-hard phase Fe_5_Y, and magnetically hard phase Y_2_Fe_14_B. In the case of the FeNbYB alloy, an axial shape of the hysteresis loop was observed, indicating the appearance of a magnetic phase in the alloys with hard or semi hard magnetic properties.

The magnetizing process of all the tested alloys in high magnetic fields is associated with the presence of pseudodislocation dipoles—whose dimensions do not exceed the exchange distance. 

According to H. Kronmuller’s theory, at a given range of external magnetic field strength, only one type of defect affects magnetization, in order: point defects, and linear defects satisfying the relationship D_dip_ < 1_H_ and D_dip_ > 1_H_. At higher values of external magnetic field intensity, magnetization is associated with the damping of thermally excited spin waves. The transition between the subsequent stages of the magnetization process must be smooth, that is, if linear defects satisfying the D_dip_ <1_H_ relationship affect the magnetization process in the range of 0.2 T, then above this area the D_dip_ > 1_H_ relationship must be satisfied—or the Holstein–Primakoff paraprocess must occur. 

Due to the presence of linear defects satisfying the relationship D_dip_ < 1_H_, it is possible to determine such parameters as the dimensions of the defects (dipole width D_dip_) and the surface density of N_dip_ linear defects.

The results presented in Table 1 confirm that the increasing Y content in the alloy causes the decrease surface density of linear defects, N_dip_ (for alloys with Hf). This, in turn, increases the packing density of atoms. For a sample without the Hf addition, the width of the pseudodislocation dipole is the largest, which causes a decrease in the D_spf_ value.

For each of the examined alloys, the magnetization process in strong magnetic fields (above the range where the relationships involving the influence of structural defects are valid) proceeded as a result of Holstein–Primakoff paraprocess, i.e., by damping of the thermally induced spin waves by the magnetic field.

The existence of phases within the amorphous structure and the creation of clusters of the Fe_5_Y crystalline phase affect changes in the interactions between the magnetic atoms. Inhomogeneity of the structure hinders the movement of the spin-wave and causes a decrease in the value of the D_spf_ parameter. An increase in the coercivity value for the Fe_65_Nb_5_Y_10_B_20_ alloy is also connected with the existence of different phases within the structure. Creation of crystalline phase nuclei prevents movement of the domain walls and hinders the magnetization process. 

**Figure 10:**
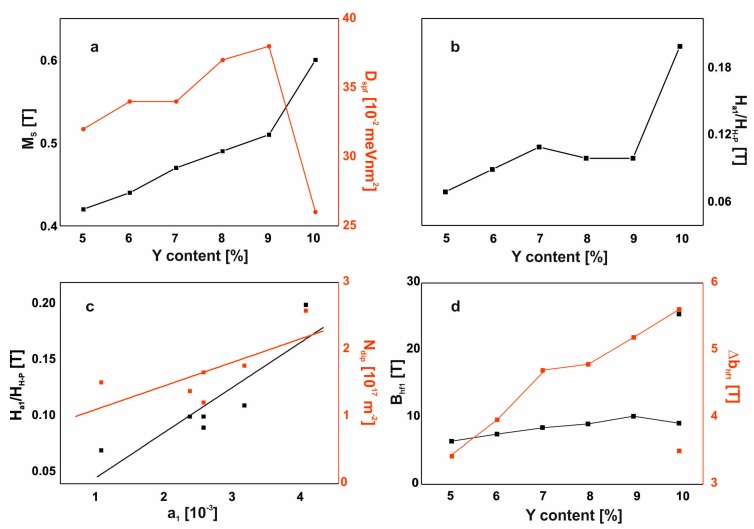
Dependence of magnetization process parameters on the chemical composition of the alloy: (**a**) M_s_ and D_spf_ in Y content function, (**b**) transition field to paraprocess in Y content function, (**c**) dependence transition field to paraprocess and N_dip_ on the factor a_1_, (**d**) B_hf1_ and ΔB_hf1_ in Y content function.

Based on the test results, some relationships were found. They are presented in the form of a diagram in Figure 10.

The first relationship found is the monotonic increase in saturation magnetization with increasing Y content in the alloy (an increase of approximately 0.02T per 1 atomic% Y). The exception in this case is the alloy with no addition of Hf, for which the saturation magnetization increases by 0.09 T. A similar relationship was found for the value of the transition field to the Holstein–Primakoff paraprocess. Based on these relationships, it should be stated that the examined process of magnetizing the examined alloys is identical for all the tested Y contents—from 5% to 9% atomic. For these alloys, the value of the D_spf_ parameter increases linearly. In the case of the alloy without the addition of Hf, significant structural reconstruction occurs. This alloy is harder to magnetize, as evidenced by the higher value of the transition field to the Holstein–Primakoff paraprocess and the much higher value of the coercive field, compared to other alloys. It is also interesting that this alloy has the highest M_S_ and the lowest value of the D_spf_ parameter. The value of this parameter can be associated with the environment of the Fe atoms: the number of magnetic neighbors and the distances between them. In fact, this part of the work is not provided with literature equivalents and one can get the impression that the information presented is based on assumptions. According to Kaul [29] and Corb [30] in the work of Kronmüller, in a relaxed amorphous structure, each magnetic atom has 12 neighbors, and in non-relaxed, 9–10 neighbors. An increase in the D_sp_ parameter value may be associated with an increased number of the nearest magnetic atoms, which is associated with the improvement of short-range chemical order (SRO). A lower value of the D_spf_ parameter in this case may mean an increase in the distance between individual Fe atoms. The arrangement of atoms in this alloy can reduce the presence of antiferromagnetic ordering, resulting in a higher saturation magnetization. However, the configuration of magnetic atoms in the Fe_65_Nb_5_Y_10_B_20_ alloy has a negative effect on creating soft magnetic properties, causing a significant increase in the coercive field value. The dependence of the coefficient *a_1_* on the value of the transition field to the Holstein–Primakoff paraprocess was also identified: as the coefficient a1 increases, the necessary value of the external magnetic field—in which magnetization depends on the damping of thermally-induced spin-waves—also increases. A similar relationship was found for the density of linear defects: N_dip_ increases with increasing value of the coefficient a_1_.

## 5. Conclusions

In this paper, the structure and process of magnetization of rapidly quenched, iron-based bulk alloys were tested. All of the tested alloys were characterized by an amorphous structure. Based on analysis of the primary magnetization curves, it was found that the process of magnetizing these materials in high magnetic fields was associated with the rotation of the magnetization vector around pseudodislocation dipoles. These defects did not, in any case, exceed the dimensions of the exchange distance. Despite the large similarities in magnetic properties, it should be noted that the addition of Y, at the expense of Hf, had a significant impact on the structure of the alloys tested. As shown in this work, the chemical composition of the alloy affected the distribution of magnetic atoms within the volume of the alloy—and the creation of the magnetic properties exhibited by the alloys. Based on analysis of the results, it was found that:

- An increase in the Y content (from 5% to 9%), at the expense of Hf (from 5% to 1%), caused an increase in the saturation magnetization value and the value of the D_spf_ parameter, which indicated the stabilizing role of Y and a similar configuration of magnetic atoms in the volume of the alloys;
- A low M_S_ value, in relation to the Fe content in the alloy, may be associated with the presence of an anti-ferromagnetic structure;
- On the basis of analysis of the Mössbauer spectra, it can be stated that the introduction of Y, at the expense of Hf, affects the chemical and topological ordering connected indirectly with the magnetic structure. The distributions of the hypermagnetic fields for the five alloys were combined from the ferro- and paramagnetic phases (97% and 3% respectively). In the alloy with no Hf content (Y at 10 at. %), there was destabilization of the amorphous matrix and separation of clearly visible amorphous phases and a single phase with an order similar to the Fe_5_Y phase;
- A different degree of disordered structure resulted in an increase in the saturation magnetization and a difficult magnetization process, which was confirmed by the high value of the coercive field and the highest value of the transition field to the Holstein–Primakoff paraprocess;
- Factor *a*_1_, determining the presence of linear defects that satisfy the relationship D_dip_<1_H_, was related to the density of N_dip_ defects and the value of the transition field to the H_a1_/H_H-P_ paraprocess.

## Figures and Tables

**Table 1:** Parameters determined on the basis of primary magnetization curve analysis.

alloy	*a*_1_	*H*_*a*1_/*H*_*H-P*_	*b*	*D_spf_*	*N_dip_*	*M_S_*	*H_c_*
Fe_65_Nb_5_Y_5_Hf_5_B_20_	1.1	0.07	0.094	32	1.51	0.42	58
Fe_65_Nb_5_Y_6_Hf_4_B_20_	2.8	0.09	0.086	34	1.66	0.44	45
Fe_65_Nb_5_Y_7_Hf_3_B_20_	3.2	0.11	0.085	34	1.76	0.47	20
Fe_65_Nb_5_Y_8_Hf_2_B_20_	2.0	0.1	0.077	37	1.38	0.49	13
Fe_65_Nb_5_Y_9_Hf_1_B_20_	2.6	0.1	0.072	38	1.21	0.51	35
Fe_65_Nb_5_Y_10_B_20_	4.1	0.2	0.128	26	2.58	0.60	1170

## Supplementary Materials

The following are available online at https://www.mdpi.com/1996-1944/13/6/1367/s1, Magnetic measurements were carried out using a VSM vibration magnetometer. The measurement accuracy is confirmed by the static magnetic hysteresis loop measured without a mounted sample included in the Supplementary materials.
